# Acid- and Volume-Sensitive Chloride Currents in Microglial Cells

**DOI:** 10.3390/ijms20143475

**Published:** 2019-07-15

**Authors:** Michael Kittl, Katharina Helm, Marlena Beyreis, Christian Mayr, Martin Gaisberger, Martina Winklmayr, Markus Ritter, Martin Jakab

**Affiliations:** 1 Institute of Physiology and Pathophysiology, Paracelsus Medical University, 5020 Salzburg, Austria; 2 Department of Biosciences, University of Salzburg, 5020 Salzburg, Austria; 3 Ludwig Boltzmann Institute for Arthritis and Rehabilitation, Paracelsus Medical University, 5020 Salzburg, Austria; 4 Gastein Research Institute, Paracelsus Medical University, 5020 Salzburg, Austria

**Keywords:** acidic, anion, chloride, Cl^−^ current, microglia, pH, RVD, swelling-activated, volume regulation

## Abstract

Many cell types express an acid-sensitive outwardly rectifying (ASOR) anion current of an unknown function. We characterized such a current in BV-2 microglial cells and then studied its interrelation with the volume-sensitive outwardly rectifying (VSOR) Cl^−^ current and the effect of acidosis on cell volume regulation. We used patch clamp, the Coulter method, and the pH-sensitive dye BCECF to measure Cl^−^ currents and cell membrane potentials, mean cell volume, and intracellular pH, respectively. The ASOR current activated at pH ≤ 5.0 and displayed an I^−^ > Cl^−^ > gluconate^−^ permeability sequence. When compared to the VSOR current, it was similarly sensitive to DIDS, but less sensitive to DCPIB, and insensitive to tamoxifen. Under acidic conditions, the ASOR current was the dominating Cl^−^ conductance, while the VSOR current was apparently inactivated. Acidification caused cell swelling under isotonic conditions and prevented the regulatory volume decrease under hypotonicity. We conclude that acidification, associated with activation of the ASOR- and inactivation of the VSOR current, massively impairs cell volume homeostasis. ASOR current activation could affect microglial function under acidotoxic conditions, since acidosis is a hallmark of pathophysiological events like inflammation, stroke or ischemia and migration and phagocytosis in microglial cells are closely related to cell volume regulation.

## 1. Introduction

Strong extracellular acidification induces an acid-sensitive outwardly rectifying (ASOR) anion current/channel in many cell types, which is also known as PAORAC (proton-activated, outwardly rectifying anion currents), or ICl_acid_/I_Cl(H)_/I_Cl(pHac)_ (acid-activated Cl^−^ currents). The biophysical and pharmacological profile of this current is remarkably similar in different cell types. Its activation threshold is around pH 5.0, it is strongly outwardly rectifying, shows time-dependent activation at constant positive membrane potentials (V_mem_), maintains an ion permeability sequence of SCN^−^ > I^−^ > NO_3_^−^ > Br^−^ > Cl^−^, and it is blocked by a wide range of unspecific Cl^−^ channel blockers such as DIDS, NBBP, niflumic acid, or phloretin [1,2,3,4,5,6,7,8,9,10,11,12,13,14].

Some of these properties, like outward rectification, ion permeability sequence, and blocker sensitivity, are similar to the ubiquitously expressed and extensively characterized volume-sensitive outwardly rectifying (VSOR) anion (Cl^−^) current, which is also known as volume-regulated anion current (VRAC), swelling-dependent Cl^−^ current (ICl_swell_), volume-sensitive organic osmolyte and anion current (VSOAC), or regulatory volume decrease current (RVDC). The current is slowly activated by osmotic cell swelling or early in apoptosis and it is substantially involved in regulatory volume decrease (RVD) and apoptotic volume decrease (AVD), respectively, and cell volume-related processes, like cell proliferation, migration, and phagocytosis [15,16,17,18,19,20,21,22,23]. While the role of VSOR channels in cell volume homeostasis as efflux pathway for anions and osmolytes is increasingly understood, functions of the ASOR current are less obvious, given that massive acidification is required to activate the conductance. E.g., an involvement in acidotoxic necrotic cell death, bone reabsorption, and spermatogenesis has been suggested in epithelial HeLa cells and mouse cortical neurons [9,10], osteoclasts [7,8], and Sertoli cells [1], respectively.

Microglial cells are resident innate immune cells of the central nervous system. Under physiological conditions, they show a ramified morphology with a small soma and thin processes (resting state). The cells undergo morphological changes by retracting their processes and increasing cell size upon the detection of neuronal damage, nervous system dysfunctions, or brain lesions. In the activated state, they migrate to the site of brain injury and phagocytose affected cells and cell fragments [24]. Migrating microglial cells undergo cell polarity-dependent cell volume changes through repetitive cycles of protrusion at the cell front (leading edge or lamellipodium) and the retraction of the cells’ rear (trailing) edge. In terms of cell volume regulation, this can be described as an alternating cycle of regulatory volume increase (RVI) at the leading edge and RVD at the trailing edge, along with cytoskeletal rearrangements. The efflux of K^+^ and Cl^−^ ions accomplishes RVD in the rear part and the VSOR currents play a key role in this process [18,20]. In microglial cells, the VSOR currents are not only involved in migration, but also in particle engulfment during phagocytosis [21,25,26,27,28]. Current inhibition has been shown to prevent the uptake of immunoglobulin-coated microspheres and disrupt the formation of lamellipodia [21,25]. Moreover, chemotaxis and particle engulfment by immune cells is impaired under hypertonic conditions [20,29], showing that migration and phagocytosis are tightly coupled to cell volume regulation.

A role of the ASOR current in these processes is elusive. In the case of ischemia, inflammation, tumor, or traumata, when pH regulating mechanisms fail to maintain a constant pH, the acidotoxic environment causes the swelling of neurons and glial cells, which poses a challenge to the cell volume regulatory mechanisms [30,31,32,33]. Under such conditions, ASOR current activation could affect the microglial function. Therefore, the aim of this study was to biophysically and pharmacologically characterize the ASOR current in BV−2 microglial cells, with special emphasis on its interrelation with the VSOR current and the effect of extracellular acidification on the cell volume regulatory ability.

## 2. Results

### 2.1. Current Activation by Extracellular Acidification, Current Amplitudes and Phenotypes

**Figure 1:**
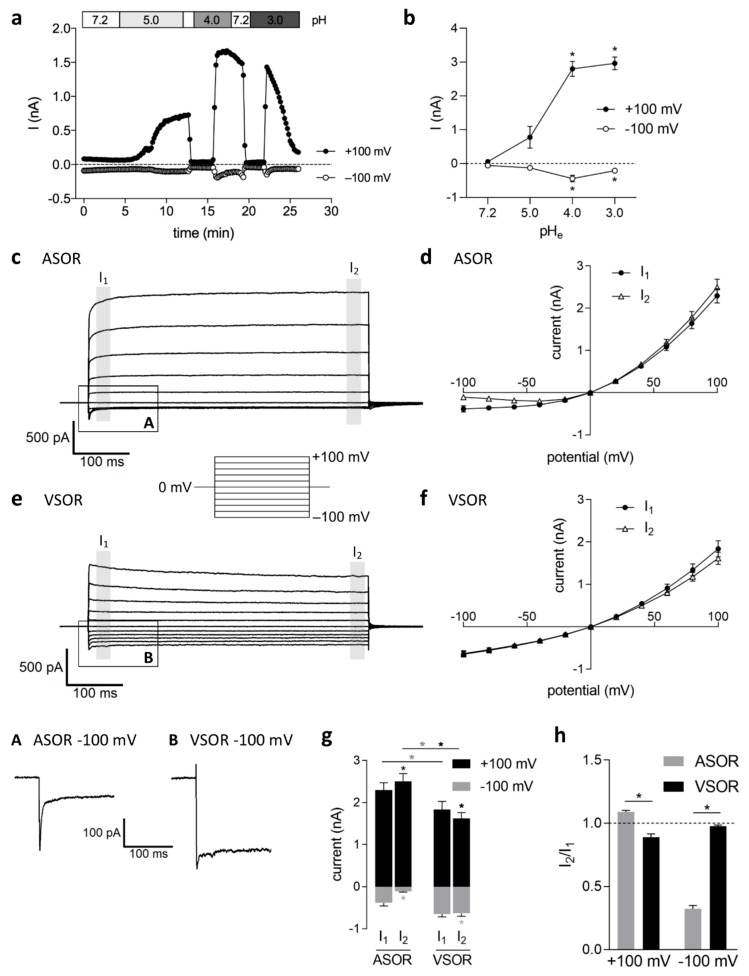
Activation kinetics and biophysical properties of the acid-sensitive outwardly rectifying (ASOR) and volume-sensitive outwardly rectifying (VSOR) current in BV-2 microglial cells: (**a**) Time course of current activation by extracellular acidification (pH 5.0, 4.0 and 3.0) at +100 (black circles) and −100 mV (empty circles); (**b**) Mean values ± standard error of the means (SEM) of currents measured at pH 7.2, 5.0, 4.0 and 3.0 (*n* = 6–12). Asterisks indicate significance compared to pH 7.2 (* *p* < 0.05); (**c**) ASOR currents elicited by 500-ms voltage steps from −100 to +100 mV in 20-mV increments (holding potential 0 mV). Expansion A: transient inward current peak at −100 mV; (**d**) ASOR current amplitudes (means ± SEM; *n* = 18) analyzed at the beginning (I_1_) and at the end (I_2_) of the voltage pulses (grey shadings); (**e**) VSOR currents recorded as in c. Note the lacking initial inward current peak at −100 mV (expansion B) as compared to the ASOR current; (**f**) VSOR current-voltage relation (means ± SEM; *n* = 9) analyzed as in d; (**g**) Maximum ASOR and VSOR current amplitudes at +100 mV (black bars) and −100 mV (grey bars). Data are identical to the values at +100 and −100 mV depicted in d and f. Black and grey asterisks indicate significant differences at +100 and −100 mV, respectively (* *p* < 0.05); (**h**) I_2_/I_1_ ratios of ASOR (grey bars) and VSOR (black bars) at +100 and −100 mV (I_2_/I_1_ > 1, time-dependent activation; I_2_/I_1_ < 1, time-dependent inactivation) (* *p* < 0.05).

The baseline whole-cell currents were low in patch clamp recordings that started under isotonic conditions and an extracellular pH of 7.2. Reducing the pH to 5.0 or lower elicited a strongly outwardly rectifying anion current. Activation was slower and gradual at pH 5.0 and was steeper at lower pH values (4.0 and 3.0; Figure 1a). The time span to the onset of current activation varied between the experiments from < 1 min. up to 10 min. Under pH 4.5 and lower, the current activated to maximum amplitudes within seconds, but then gradually inactivated over time, which was particularly visible at pH 3.0 (Figure 1a). At pH 2.0, the current rapidly, but only transiently, appeared, while the cells already developed signs of acidotoxic damage after a short time under these extreme conditions. Therefore, we avoided more acidic conditions than pH 3.0 and most series were performed at pH 4.5 in all subsequent experiments. Figure 1b shows the average current amplitudes measured under pH 7.2 (control; *n* = 6), 5.0, 4.0, and 3.0 (*n* = 12) with half-maximal current activation at a pH of ~5.3.

The pH dependency of activation and current kinetics were identical to the acid-sensitive outwardly rectifying (ASOR) Cl^−^ currents that were described in other cell types [1,2,3,4,5,6,7,8,9,10,11,12,13,14]. The current showed facilitation over time at constant positive holding potentials and an initial negative current peak at −100 mV (Figure 1c and trace expansion A). The currents were analyzed at the beginning and at the end of the 500-ms voltage pulses (I_1_ and I_2_, respectively). The mean ASOR current amplitudes recorded at pH 4.5 were 2.30 ± 0.17 nA (I_1_) and 2.50 ± 0.19 nA (I_2_) at +100 mV and −0.39 ± 0.08 nA (I_1_) and −0.11 ± 0.02 nA (I_2_) at −100 mV (*n* = 18) (Figure 1d,g) and they displayed time-dependent activation over time at +100 mV (*p* < 0.0001) and current inactivation at −100 mV (*p* < 0.001) at constant holding potentials (Figure 1g). This is also evident from the I_2_/I_1_ ratios (> 1.0 at +100 mV and < 1.0 at −100 mV, respectively) in Figure 1h. The ASOR current rapidly reached stable peak amplitudes from the onset of activation under pH 4.5 or lower.

The volume-sensitive outwardly rectifying (VSOR) Cl^−^ current, which we have previously characterized in BV-2 cells [21,27], developed more slowly over time, reaching an activation plateau after 10–20 min. VSOR currents that were activated by an 80 mOsm/kg reduction in extracellular osmolality under pH 7.2 showed a typical morphology known from many cell types [17,23] (Figure 1e). Mean VSOR current amplitudes at +100 mV were higher at I_1_ (1.84 ± 0.19 nA) than at I_2_ (1.62 ± 0.14 nA) (*p* < 0.05; *n* = 9), which indicated time-dependent inactivation at constant positive holding potentials, which is phenotypical to this current (Figure 1e–g). This inactivation is also reflected by an I_2_/I_1_ ratio < 1.0 (Figure 1h). VSOR current amplitudes at −100 mV −0.65 ± 0.07 nA (I_1_) only slightly, though significantly (*p* < 0.01), declined over time to −0.63 ± 0.07 nA at I_2_ (*n* = 9; Figure 1g), yielding an I_2_/I_1_ ratio of ~1.0 (Figure 1h). Overall, outward ASOR current amplitudes in BV-2 cells were significantly higher when compared to VSOR currents (*p* < 0.01), while VSOR currents were higher in the inward direction (*p* < 0.05) (Figure 1g).

### 2.2. Relative Ion Permeability of the ASOR Current and Blocker Sensitivity

**Figure 2:**
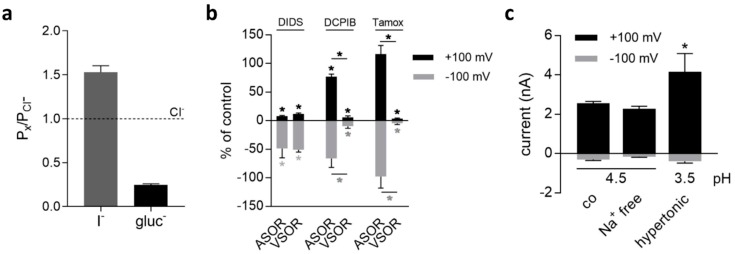
(**a**) ASOR current anion permeability in BV-2 cells (gluc^−^, gluconate^−^); (**b**) Pharmacology of ASOR and VSOR currents. Currents at +100 (black bars) and −100 mV (grey bars) in presence of DIDS (100 µM), DCPIB (10 µM), or tamoxifen (Tamox; 10 µM) in% of current amplitudes measured in the absence of inhibitors. Black and grey asterisks indicate significant differences at +100 and −100 mV, respectively (* *p* < 0.05); (**c**) ASOR current activation under Na^+^ free (*n* = 3) and hypertonic conditions (350 mOsm/kg; *n* = 3) as compared to control (co) currents at pH 4.5 (300 mOsm/kg; *n* = 26). Mean values ± SEM (* *p* < 0.05).

Cl^−^ in the bath solution was replaced with equimolar concentrations of iodide (I^−^) or gluconate to determine the relative anion permeabilities of the ASOR current. In the presence of I^−^ and gluconate, the reversal potential (E_rev_) shifted by −9.94 ± 1.16 mV (*p* < 0.001) and 29.77 ± 0.10 mV (*p* < 0.01), respectively. Relative permeabilities, as calculated from the shifts in E_rev_ while using the Goldman-Hodgkin-Katz equation, were I^−^ : Cl^−^ : gluconate^−^ = 1.53 ± 0.07 : 1 : 0.25 ± 0.01 (*n* = 3–7; Figure 2a).

We used the Cl^−^ channel inhibitors DIDS, DCPIB, and tamoxifen for pharmacological profiling of ASOR and VSOR currents. While DIDS blocks Cl^−^ transport mechanisms with low specificity, DCPIB, and tamoxifen are more specific for swelling-activated (VSOR) Cl^−^ currents [23]. As shown in Figure 2b, DIDS (100 µM) significantly inhibited both conductances to approximately the same extent in a voltage-dependent manner. ASOR and VSOR outward currents at +100 mV were blocked to 7.9 ± 1.3% (*n* = 6; *p* < 0.001) and 12.0 ± 1.5% (*n* = 4; *p* < 0.05), inward currents at −100 mV to 48.7 ± 16.5% (*n* = 6; *p* < 0.05), and 50.9 ± 4.1% (*n* = 4; *p* < 0.05) of control values, respectively. In contrast to DIDS, the currents showed significantly different sensitivities to DCPIB and tamoxifen. The ASOR current was far less sensitive with an inhibition to 76.9 ± 4.7% (*n* = 6; *p* < 0.01) and 66.2 ± 15.4% (*n* = 6) while the VSOR current was strongly inhibited by 10 µM DCPIB to 5.8 ± 2.4% (*n* = 3; *p* < 0.05) and 9.7 ± 3.6% (*n* = 3; *p* < 0.05) at +100 and −100 mV. Moreover, the ASOR current was completely insensitive to 10 µM tamoxifen, a concentration which caused an almost complete block of the VSOR outward and inward current to 3.4 ± 1.3% (*n* = 3; *p* < 0.05) and 4.8 ± 1.9% (*n* = 3; *p* < 0.05) of control, respectively.

The acid-sensitive current could be readily activated under extracellular Na^+^-free, as well as hypertonic (350 mOsm/kg) conditions. The higher outward current amplitude (*p* < 0.001) under hypertonicity can be explained by the larger step in pH to 3.5 as compared to the controls (pH 4.5). Further, the current could be activated in the ruptured patch clamp configuration and in the presence of the membrane permeable Ca^2+^ chelator BAPTA-AM, which indicated that an intact intracellular compartment and intracellular Ca^2+^ ions are not prerequisite for current activation.

### 2.3. ASOR Current Activation Requires Extracellular, but not Intracellular Acidification

**Figure 3:**
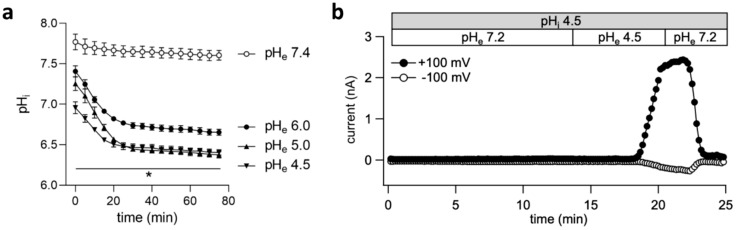
ASOR current activation is dependent on extracellular, but not intracellular acidification: (**a**) Time courses of the intracellular pH (pH_i_) in response to extracellular acidification (pH 6.0, 5.0 or 4.5; full symbols) compared to pH 7.4 (empty circles). Means ± SEM (*n* = 3). At any time point the values at pH 6.0, 5.0 and 4.5 were significantly different from values measured at pH 7.4 (* *p* < 0.05–0.0001); (**b**) Time course of the ASOR current in a BV-2 cell at +100 and −100 mV (black and grey circles, respectively) recorded in the ruptured patch clamp configuration using a pipette solution at pH 4.5 (pH_i_ 4.5; grey bar).

We monitored the intracellular pH (pH_i_) over time using the membrane permeable pH-sensitive dye BCECF-AM to test how far extracellular acidification transfers to the intracellular milieu. The pH_i_ significantly dropped to pH 6.8–6.5 within 20 min. and remained relatively stable 0.6–2 pH units above the prevailing extracellular pH over the next 55 min. after reducing the extracellular pH to 6.0, 5.0, or 4.5 (Figure 3a). This drop of pH_i_ as a result of extracellular acidification might be sensed by the ASOR channels and stimulate current activation. We performed patch clamp experiments in the ruptured configuration with a pH-4.5 pipette solution to find out whether the current can be induced by intracellular acidification. As shown in Figure 3b, ASOR currents did not activate unless the extracellular pH was reduced to 4.5. Switching back to pH 7.2 led to rapid and complete current inactivation.

### 2.4. Temperature-Sensitivity of ASOR and VSOR Currents

**Figure 4:**
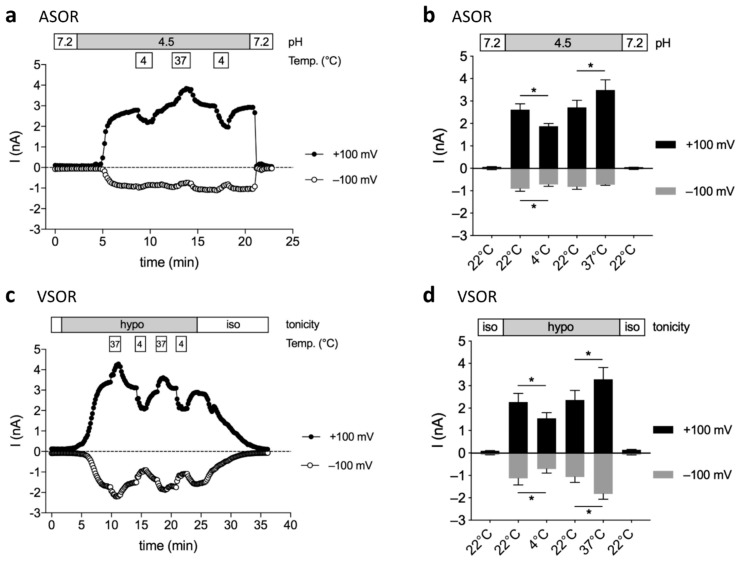
Temperature sensitivity of ASOR and VSOR currents in BV-2 cells: (**a**,**c**) ASOR and VSOR current time courses at +100 (full circles) and −100 mV (open circles) at room temperature (~22 °C), 4 °C and 37 °C. ASOR and VSOR currents were activated by pH 4.5 or hypotonicity (220 mOsm/kg), respectively; (**b**,**d**) Means ± SEM of ASOR (*n* = 3) and VSOR (*n* = 4) currents at +100 (grey bars) and −100 mV (black bars) (* *p* < 0.05).

We performed recordings at room temperature (22 °C) and after superfusion with a 4 °C and 37 °C bath solution to test for temperature effects on ASOR and VSOR currents. As shown in Figure 4a and b, at 4 °C the outward and inward ASOR currents at +100 and −100 mV were reduced by 27.8 ± 3.4% and 20.3 ± 1.4%, respectively (*n* = 3; *p* < 0.05). Warming to 37 °C increased the outward current by 28.2 ± 2.8% (*n* = 3; *p* < 0.05), while the inward current was not significantly affected (9.5 ± 9.8% reduction; *n* = 3). The VSOR outward and inward currents were diminished by 31.6 ± 2.1% and 36.4 ± 3.6%, respectively (*n* = 4; *p* < 0.05) at 4 °C (Figure 4c,d), which were comparable to the effect on the ASOR current. However, in contrast to the ASOR current, warming led to an increase not only of the outward VSOR current at +100 mV by 40.8 ± 5.4% (*n* = 4; *p* < 0.01), but also of the inward current at −100 mV by 79.7 ± 18.5% (*n* = 4; *p* < 0.05).

### 2.5. Transient Coactivation of ASOR and VSOR Currents in BV-2 cells

**Figure 5:**
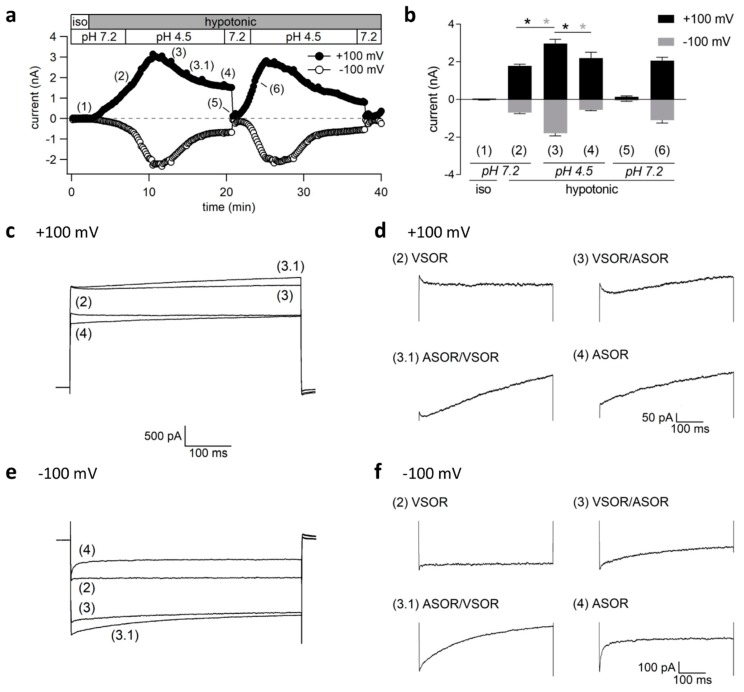
Transient coactivation of VSOR and ASOR currents in BV-2 cells: (**a**) Cl^−^ current time course of a single experiment under isotonic (iso; 300 mOsm/kg) and hypotonic (220 mOsm/kg) conditions at pH 7.2 or 4.5 as indicated. Currents were elicited by voltage ramps from −100 to +100 mV (duration 500 ms, 10-s intervals) from a holding potential of 0 mV. Currents at +100 (black circles) and −100 mV (gray circles); (**b**) Mean current amplitudes ± SEM (*n* = 5–7) measured at the time points (1)–(6) indicated in a. Black and grey asterisks indicate significant differences at +100 and −100 mV, respectively (* *p* < 0.05); (**c**,**e**) Current traces in response to 500-ms voltage steps to +100 and −100 mV, respectively, at the time points marked in a. The holding potential between steps was 0 mV; (**d**,**f**) The same traces as shown in c and e, but with extended *y*-axis spreading (note different *y*-axis scaling of traces at +100 and −100 mV).

Until this point, we either stimulated the ASOR or the VSOR current by acidification or hypotonicity, respectively. We first elicited the VSOR current in a next series of experiments, and then simultaneously exposed the cells to pH 4.5 to test whether both currents could be simultaneously active. The currents were recorded every 10 s in response to voltage ramps from −100 to +100 mV, as shown in Figure 5a. Mean current amplitudes measured at the time points (1)–(6) are shown in Figure 5b (*n* = 5–7). We applied 500-ms voltage steps from 0 mV to +100 and −100 mV at these time points to analyze the current inactivation/activation behavior at constant holding potentials (Figure 5c,e). The traces in Figure 5d,f are the same as in c and e, except for an extended *y*-axis spreading to better visualize the current kinetics.

Switching from isotonic to hypotonic conditions at pH 7.2 was followed by slow VSOR current activation from the baseline current levels to 1.79 ± 0.08 nA and −0.70 ± 0.06 nA (*n* = 7) at +100 and −100 mV, respectively (Figure 5b), with typical inactivating kinetics at +100 mV (traces (2) in Figure 5c,d). The currents significantly increased further to 2.97 ± 0.23 nA at +100 mV (*n* = 7; *p* < 0.01) and −1.79 ± 0.15 nA (*n* = 7; *p* < 0.001) at −100 mV upon simultaneous acidification to pH 4.5. As visible in the traces (3) and (3.1), the whole-cell Cl^−^ current showed distinctive kinetics at +100 mV, with an initial inactivation phase followed by current facilitation over time characteristic for the ASOR current (Figure 5c,d). The higher current amplitudes and mixed kinetics indicate that VSOR and ASOR currents coexist during a limited period, giving rise to a mixed and superimposed phenotype. However, the ASOR current became the dominating conductance under continued acidity and hypotonicity, as evident from traces (4) at +100 mV. For the inward current at −100 mV the phenotype transition from an exclusive VSOR to a pure ASOR current with an initial inward current peak and progressive time-dependent inactivation is also apparent (Figure 5e,f). The amplitudes of the dominating ASOR current were significantly smaller as during the phase of current superimposition (2.20 ± 0.31 nA; *n* = 6; *p* < 0.01 and −0.55 ± 0.05 nA; *n* = 6; *p* < 0.001 at +100 and −100 mV), which indicated that the volume-sensitive current inactivated under lasting acidic conditions. Stepping back from pH 4.5 to 7.2 under continued hypotonicity caused rapid ASOR deactivation (time point (5)), while the VSOR current slowly recovered from acidic inhibition to reach amplitudes of 2.07 ± 0.17 nA at +100 mV and −1.10 ± 0.15 nA (*n* = 5) at −100 mV at time point (6).

**Figure 6:**
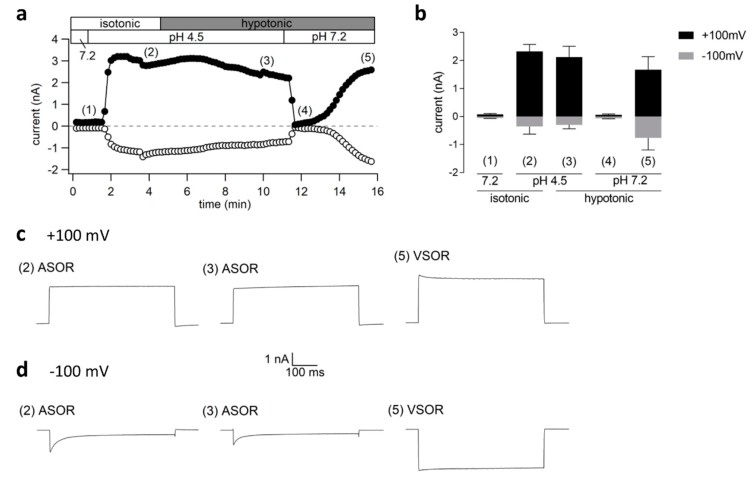
(**a**) Cl^−^ current time course of a single experiment under isotonic (300 mOsm/kg) and hypotonic (220 mOsm/kg) conditions at pH 4.5 or 7.2. Voltage ramps from −100 to +100 mV (duration 500 ms, 10-s intervals) were applied from a holding potential of 0 mV. Currents at +100 (black circles) and −100 mV (gray circles); (**b**) Mean currents ± SEM (*n* = 3–4) measured at time points (1)–(5) indicated in a; (**c**,**d**) Current traces in response to 500-ms voltage steps to +100 and −100 mV, respectively, at the time points marked in a. The holding potential between steps was 0 mV.

In the reverse approach, when first the ASOR current was activated by acidification to pH 4.5 and the cells were then simultaneously exposed to hypotonicity, we did not observe superimposed, additive currents, but a pure ASOR current phenotype (Figure 6a,b and traces (2) and (3) in c,d). The current amplitudes at +100 and −100 mV were 2.32 ± 0.26 nA and −0.36 ± 0.27 nA (*n* = 4) under pH 4.5, and 2.13 ± 0.39 nA and −0.30 ± 0.14 nA (*n* = 4; *p* > 0.5) under additional hypotonic exposure. This implies that VSOR current activation is prevented at low pH. However, stepping back to pH 7.2 under persisting hypotonicity again caused rapid ASOR current inactivation and slow VSOR current activation to 1.68 ± 0.46 nA and −0.77 ± 0.43 nA (*n* = 3) at +100 and −100 mV with a characteristic time course and current phenotype (Figure 6a,b and traces (5) in c,d).

### 2.6. ASOR Current Activation Depolarizes the Cell Membrane Potential

**Figure 7:**
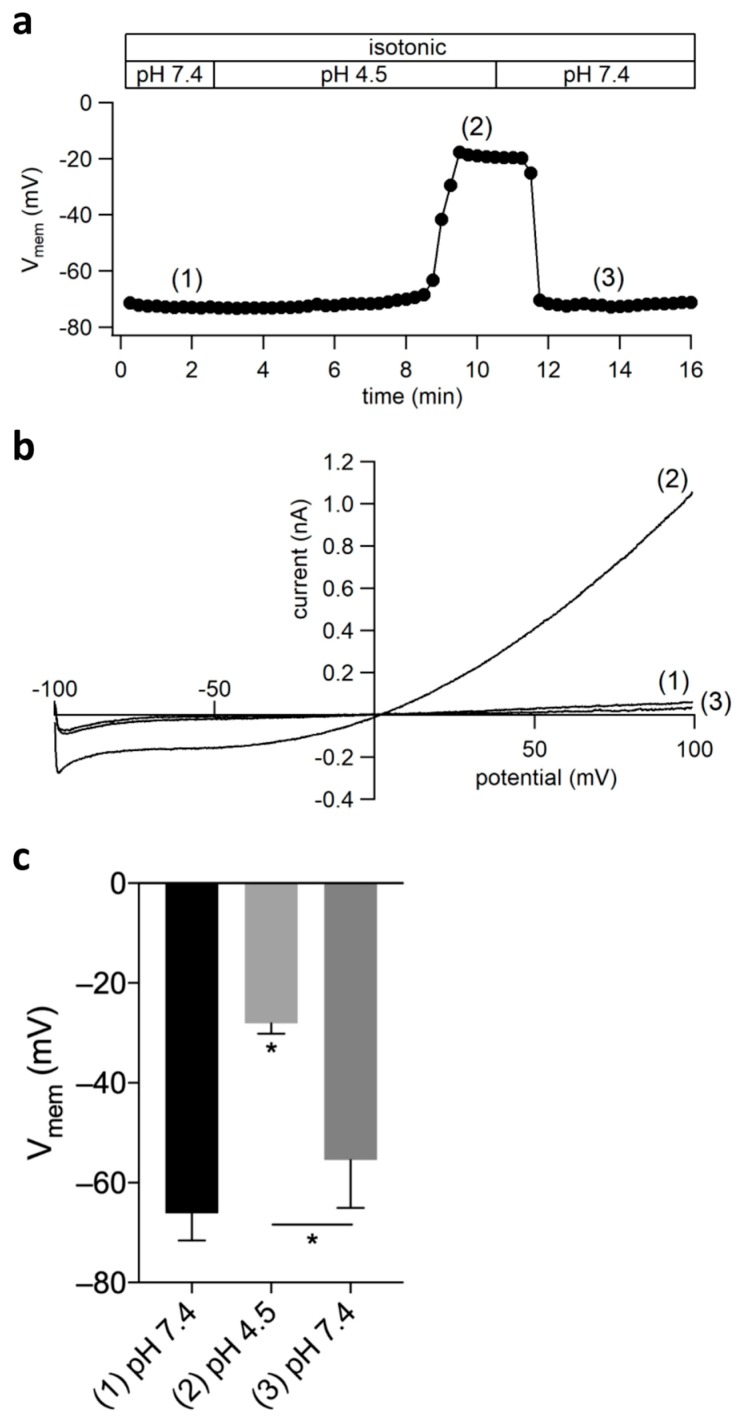
ASOR current activation depolarizes the cell membrane potential (V_mem_): (**a**) V_mem_ recording of a single BV-2 cell at pH 7.4, 4.5, and 7.4 again; (**b**) Whole-cell currents elicited by voltage ramps from −100 to +100 mV before (1), during (2), and after (3) acidity-induced depolarization; (**c**) Mean V_mem_ ± SEM of eight independent experiments as shown in a (* *p* < 0.05).

Next, we tested how far ASOR activation affects the cell membrane potential (V_mem_). Figure 7a shows a V_mem_ recording before, during, and after extracellular acidification to pH 4.5. In this experiment, with a latency of ~5 min., acidification led to a stable and reversible depolarization to ~−20 mV. We briefly switched from current to voltage clamp and applied voltage ramps to test whether the ASOR current was activated before, during, and after the acidity-induced depolarization (Figure 7b). The average resting V_mem_ under pH 7.4 was −66.13 ± 5.42 mV (*n* = 8) (Figure 7c). Under acidic conditions V_mem_ depolarized to −28.05 ± 2.07 mV (*n* = 8; *p* < 0.001). After switching back to pH 7.4 V_mem_ repolarized to −55.45 ± 9.61 mV (*n* = 8; *p* < 0.05 vs. pH 4.5).

### 2.7. Cell Volume Regulation under Acidic and/or Isotonic or Hypotonic Conditions

**Figure 8:**
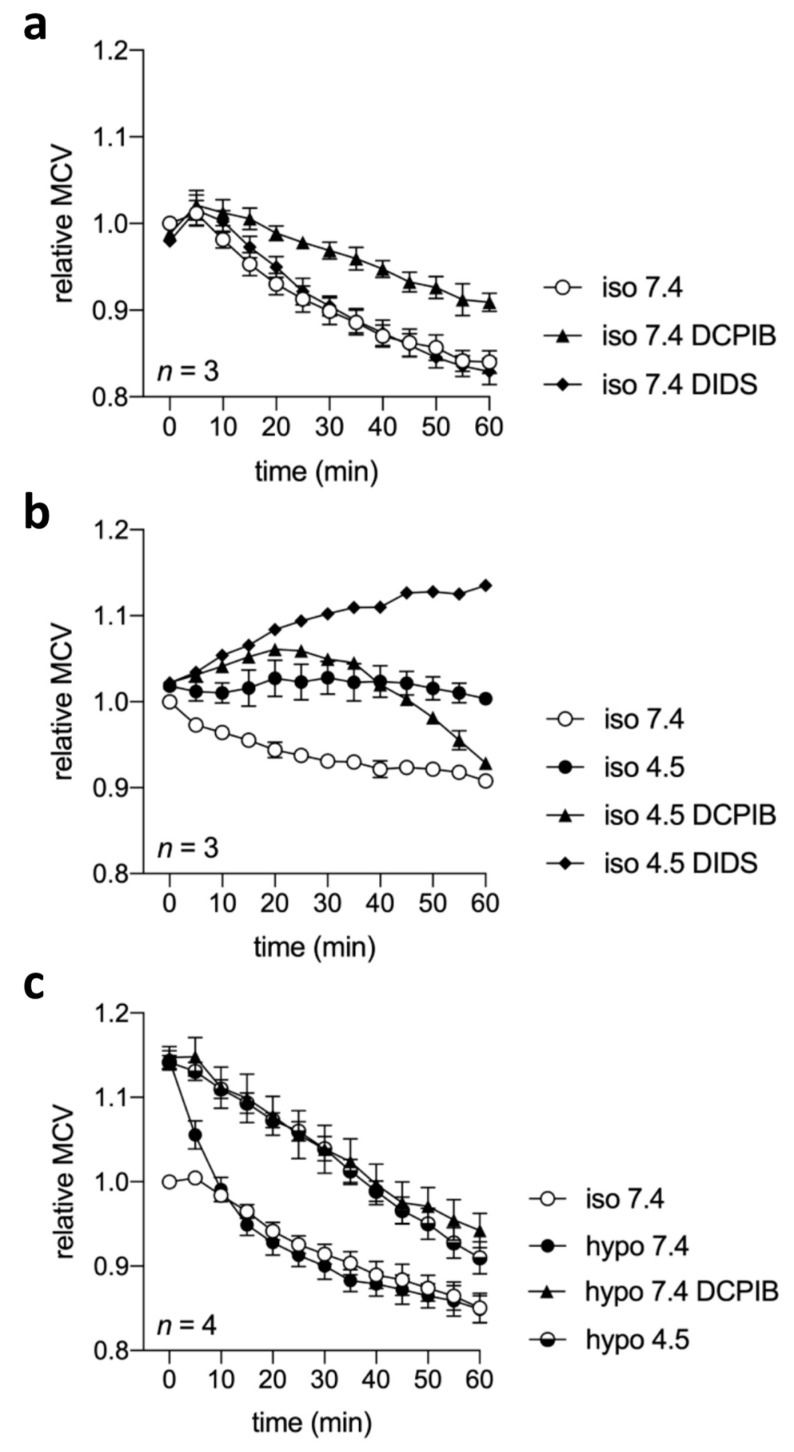
Cell volume regulation in BV-2 cells under normal pH 7.4 or acidic pH 4.5 under isotonic (iso) or hypotonic (hypo) conditions in absence or presence of Cl^−^ channel blockers: Measurements were performed using the Coulter method. Mean cell volume (MCV) over time under (**a**) iso pH 7.4 in absence or presence of DCPIB or DIDS, (**b**) iso pH 7.4 or pH 4.5 in absence or presence of DCPIB or DIDS and (**c**) iso pH 7.4, hypo pH 4.5 in absence or presence of DCPIB and hypo pH 4.5. DIDS or DCPIB were added at 10 µM. Data are shown relative to the MCV in femtoliters (fl) of the first measurement under the respective condition.

VSOR currents play a pivotal role in the regulatory volume decrease (RVD) response to counteract osmotic cell swelling [15,16,22,23]. We asked in how far cell volume regulation is affected under these conditions in view of the apparent inactivation of the VSOR current and ASOR current activation under acidic conditions. Therefore, we performed cell volume measurements under isotonic or hypotonic conditions at pH 7.4 or 4.5 in the absence or presence of the Cl^−^ channel blockers DIDS or DCPIB. We observed a 10–15% cell shrinkage from ~2 400 to ~2 100 fl over 60 min. (*n* = 10; *p* < 0.01; Figure 8a–c) under isotonic pH 7.4 (control) conditions, which was unaffected by DIDS (10 µM), but was less pronounced in the presence of 10 µM DCPIB (Figure 8a). Under isotonic conditions at pH 4.5 the shrinkage as observed at pH 7.4 was absent. In two out of three experiments, we observed cell swelling with a ~5% maximum after 20–40 min., but cells maintained a relatively constant volume over 60 min. on average. In contrast, in the presence of 10 µM DIDS, cells under pH 4.5 showed a progressive volume gain (Figure 8b). DCPIB (10 µM) had a similar effect over the first 20–25 min., but caused cell shrinkage thereafter. Cells that were exposed to hypotonic conditions under pH 7.4 accomplished a complete RVD within 10 min. from an initial ~15% swelling to ~2 750 fl (Figure 8c), which was fully inhibited by DCPIB (10 µM) and was also absent in cells that were exposed to pH 4.5.

## 3. Discussion

In this paper, we characterize an endogenous acid-sensitive outwardly rectifying (ASOR) Cl^−^ current in BV-2 microglial cells for the first time and describe its interrelation with the volume-sensitive (swelling-activated) outwardly rectifying (VSOR) Cl^−^ current. ASOR currents have been described in diverse cell types, such as epithelial cells, myocytes, osteoclasts, chondrocytes, neuronal cells, and erythrocytes [1,2,3,4,5,6,7,8,9,10,11,12,13,14,34,35]. The core characteristics of acid-sensitive Cl^−^ currents are remarkably similar. The current is activated by acidification to pH ≤ 5.0, is outwardly rectifying, and shows facilitation over time at constant positive holding potentials. In BV-2 cells, we recorded maximum ASOR current amplitudes between pH 4.0 and 3.0. The current declined again after reaching peak amplitudes at pH values < 3.0, similar to as described in HEK293 cells [3]. Outward rectification is more pronounced when compared to the VSOR current in BV-2 cells, which shows characteristic voltage-dependent inactivation over time at positive holding potentials [21,27]. Additionally, the I^−^ > Cl^−^ > gluconate^−^ anion permeability sequence, which is typical for volume-sensitive- and Ca^2+^-activated Cl^−^ channels [17,22,23], is consistent with most previous reports [2,3,4,10,11,14]. A permeability sequence characteristic for voltage-gated ClC channels (Cl^−^ ≥ I^−^ > gluconate^−^) has been reported for ASOR channels in mouse osteoclasts [7] and rat Sertoli cells [1]. In accordance with a previous study on osteoclasts [7], we show that intracellular acidification *via* a low-pH pipette solution does not activate the current, which suggests that the proton binding sites are located at the extracellular face of the plasma membrane, and that the intracellular pH does not apparently affect ASOR channel activation. This is supported by the finding that the endogenous ASOR current in HEK293 can be readily activated when using an intracellular solution that contains 100 mM HEPES [3].

Like VSOR currents, a wide range of structurally unrelated Cl^−^ transport blockers, such as DIDS, NPPB, niflumic acid, or 1,9-dideoxyforskolin, inhibits the ASOR currents [2,3,4,5,7,9,10,11,12,34]. The sensitivity of ASOR currents to DIDS is generally higher than to other inhibitors with IC_50_ values from 0.12 µM in HeLa cells [5] to 13 µM in human chondrocytes [34]. In BV-2 cells, both ASOR and VSOR currents are voltage-dependently inhibited to the same extent by 100 µM DIDS. Generally, the low specificity of most of these inhibitors, including DIDS, limits their usability in discriminating between Cl^−^ conductances in a cell system. For DCPIB and tamoxifen, which are regarded as more specific for VSOR currents [23], we found distinct differences in the blocker sensitivities. Whereas, the VSOR current was almost completely blocked by DCPIB and tamoxifen at 10 µM, the ASOR current was only weakly inhibited by DCPIB, and it was completely insensitive to tamoxifen. Similarly, DCPIB at the same concentration and tamoxifen up to 100 µM were ineffective on ASOR currents in the HeLa cells, HEK293 cells, and cardiac myocytes [2,4,12]. However, it should be considered that the inefficiency of DCPIB and tamoxifen might not primarily be due to a lack of blocker sensitivity of the channels, but due to a reduced blocker activity at strongly acidic pH.

ASOR and VSOR currents in BV-2 cells are both temperature-sensitive, however in different ways. VSOR current amplitudes were significantly higher at 37 °C than at room temperature and cooling to 4 °C had the opposite effect. To our best knowledge, this is the first report of the temperature effects on volume-sensitive Cl^−^ currents, which is surprising, given the plethora of literature on these currents. Similar to the VSOR current, the acid-sensitive current was augmented by warming and diminished by cooling. However, in contrast to the volume-sensitive current, warming only increased the outward acid-sensitive current, while the inward currents were largely unaffected. Higher outward ASOR currents at 37 °C as compared to room temperature have also been reported in HeLa cells [9] and mouse cortical neurons [10].

Extracellular acidification to pH 4.5 caused a strong depolarization of the cell membrane potential to ~−28 mV, which is above the calculated reversal potential of Cl^−^ of ~−40 mV while assuming the complete equilibration of the cytoplasmic Cl^−^ concentration with the pipette solution during the recordings. This implies that, besides ASOR currents, the activation of acid-sensitive cation conductances is likely to contribute to the depolarization. e.g., functional expression of both acid-sensing ion channels (ASICSs) [36] and acid-sensitive transient receptor potential cation channels (TRPM7) [37] has been shown in primary rat microglial cells.

Including our present work, only a few studies have been performed to investigate the interrelation between ASOR and VSOR channels/currents by combining the hypotonic- and acidic challenge. There is a controversial discussion as to whether the same or different channel entities underlie both ASOR and VSOR currents. While Nobles et al. [2] postulated that the two currents are manifestations of the same ion channel entities, Lambert and Oberwinkler [3] argued that the ASOR and VSOR channels are distinct populations of ion channels and provided evidence that they can be simultaneously active in the same cell. We observed short-term current superimposition and a mixed current phenotype when the VSOR current was first activated by hypoosmotic stimulation and cells were then simultaneously exposed to low pH. Lambert and Oberwinkler [3] found a transient decrease in the outward current immediately after exposure to pH 4.5, indicating VSOR current inhibition by acidic pH, consistent with our observation in BV-2 cells, in HEK293 cells. Thereafter, the current amplitude increased. From these observations, the authors concluded that the whole-cell current under simultaneous hypotonic and acidic conditions consists of two independent current components. However, in BV-2 cells, the current changed to a pure ASOR phenotype after a short period of time and when the ASOR current was activated first, the activation of the VSOR current under simultaneous hypotonic conditions was prevented. Therefore, we assume that strong acidification to pH 4.5 fully inhibits the VSOR current. This would also explain why we were not able to measure any Cl^−^ current immediately after switching from pH 4.5 to 7.4 under continued hypotonicity, since the volume-sensitive current first needs to be reactivated from acidic inhibition. The partial inhibition of VSOR currents and accelerated inactivation at positive potentials under less acidic conditions (pH 5.0 and higher) has previously been shown in *Xenopus* oocytes [38], C6 glioma cells [39], BC3H1 mouse myoblasts [40], mouse neuroblastoma cells [41], bovine pulmonary artery cells [42], and endothelial cells [43]. Under which conditions, for how long, or if at all ASOR and VSOR currents can coexist under hypotonic and additional acidic conditions may, amongst other factors, depend on the duration of exposure, the magnitude of the challenge, the applied pulse protocols, and the relative amplitudes of ASOR and VSOR currents in the respective cell type. We also show that the acid-sensitive current can be fully activated under hypertonic conditions, which suggests that the ASOR channel gating is unaffected by osmotic changes.

The homogeneous biophysical and pharmacological properties of ASOR currents in different cell types suggest a common molecular basis. Several studies have been performed to identify the protein(s) that form the ASOR channel. In recent years, the pannexin-related leucine-rich repeats containing 8A (LRRC8A/SWELL1) protein and its paralogs have been identified as essential VSOR channel components [44,45]. It has been speculated that these might also be involved in ASOR channel pore formation when considering similarities of the two currents in respect of outward rectification and anion selectivity. However, silencing of LRRC8 genes did not affect ASOR channel activity in HeLa cells, whereas the VSOR currents were diminished [12,13]. In HEK293 cells, siRNA knock-down of TMEM16/anoctamin family members, which are associated with Ca^2+^-activated Cl^−^ currents (CaCCs) and show certain phenotypical similarities to ASOR currents (outward rectification, activation over time at positive potentials [46]), resulted in a significantly reduced CaCC, but it had no effect on the ASOR current [11]. ASOR currents being apparently unrelated to CaCCs is also substantiated by our own finding and the observation of others [2,3,7,11], that chelating intracellular Ca^2+^ with BAPTA does not affect the current. While several studies have suggested a relation between ASOR currents and the expression of the intracellular Cl^−^ channels ClC-3 and ClC-7, e.g., in mouse osteoclasts [7,8], human nasopharyngeal carcinoma CNE-2Z cells [47] and HEK293 cells [7,35], others did not observe an effect on the endogenous ASOR currents upon siRNA-mediated ClC-3 or ClC-7 knock-down or ClC-3 overexpression in HEK293 and HeLa cells [9,11,35]. Indeed, the ASOR currents show resemblance to ClC-3/7 currents, which are, however, functional in intracellular organelles, rather than in the plasma membrane. Under normal pH, ClC-3 and ClC-7 work as Cl^−^/H^+^ antiporters, but they are uncoupled to function as selective Cl^−^ channels at acidic pH [35]. Organelles with pH values in this range (pH 4–6) are lysosomes, late endosomes, and phagosomes [48], where the ASOR current might serve to electrically counterbalance the proton influx. Regarding ASOR activity in the plasma membrane, it has been put forward that strong extracellular acidosis might cause deranged intracellular trafficking and trigger the fusion of ASOR channel-containing submembranous vesicles with the plasma membrane. This is supported by the finding that colchicine, which is an inhibitor of tubulin polymerization, caused a significant reduction in the ASOR current amplitude in the HEK293 cells [11]. Accordingly, the drop in intracellular pH in consequence of strong extracellular acidification, as we show here, would not *per se* trigger the ASOR current, but divert channel proteins into the plasma membrane, where the exposure of the channels’ proton sensors to the acidic extracellular space causes current activation. On the other hand, it still cannot be ruled out that ASOR and VSOR channels share common molecular entities. The switch between the two currents might be caused by conformational changes of the channel proteins, due to protonation/deprotonation and/or reassembly of channel components. It is also conceivable that, despite very similar current kinetics, the cell type-specific subunit compositions of ASOR channels might account for differences in the anion permeability or blocker sensitivity. Accordingly, the knock-down of a specific channel protein candidate might not necessarily mean a significant impact on the absolute current magnitude or phenotype, but rather cause changes, e.g., in the pH sensitivity, ion selectivity, or pharmacological profile. Just recently, by performing RNA interference screening, a significant step towards the identification of the molecular basis of ASOR currents has been made with the identification of TMEM206 as a core component of the ASOR channel pore [49,50], which will push the understanding of ASOR channel function in future studies.

Functions of the ASOR current, or the consequences of its activation under physiological or pathophysiological conditions, are elusive when considering the massive extracellular acidification that was required for the channels to open. Importantly, Sato-Numata et al. observed a shift in the half-maximum activation pH value of the ASOR current from 4.9 at 25 °C to 5.4 at 37 °C in mouse cortical neurons [10]. The authors suggested that the activation of the current promotes acidotoxic cell death and that its temperature sensitivity might at least in part underlie the neuroprotective effect of hypothermia under acidotoxic conditions that are associated with pathophysiologic states in the brain. A similar temperature-dependent shift in the half-maximum activation pH and a relation between ASOR current activation and acidotoxic cell death has also been described in HeLa cells [5,9], and TMEM206 knock-out HEK293 cells transiently transfected with human TMEM206 [49]. This stresses the importance of this novel class of channel proteins in ASOR pore formation. In Sertoli cells and osteoclasts, which generate an acidic environment that is necessary for sperm maturation and bone reabsorption, respectively, ASOR channels are thought to be functionally coupled to the extrusion of H^+^ ions [1,7,8]. A function in electrically counterbalancing proton efflux is also conceivable for microglial cells and phagocytes in general during the respiratory burst, when the cells get acidified intracellularly and large amounts of protons from the NADPH oxidase reaction are extruded, e.g., *via* proton channels [51]. It is relevant in this context that, in neurodegenerative states, like Alzheimer’s disease, the activation of microglial cells aggravates disease progression due to massive superoxide formation by the NADPH oxidase [52]. Proton extrusion during the oxidative burst might cause local acidification in the vicinity of the plasma membrane sufficient for ASOR current activation.

VSOR currents play a key role during RVD in counteracting osmotic cell swelling [15,16,22,23]. In microglial cells, moreover, migration and phagocytosis are tightly related to cell volume regulatory processes and they are dependent on VSOR current activity [21,25,26,27,28]. Therefore, we were particularly interested in how far cell volume regulation was affected by acidification. We observed 10–15% cell shrinkage over time under isotonic conditions and normal pH, similar to that described in other cell types under comparable experimental conditions [5,10,53]. This might be caused by an imbalance in osmolality under the given conditions with relative hypertonicity of the extracellular solution. Gradual shrinkage under nominal isotonicity was attenuated by DCPIB, which suggests that basic VSOR channel activity and the outward leakage of Cl^−^ ions and/or organic osmolytes might underlie the loss in cell volume under control conditions. That DIDS was without effect might be due to its voltage-dependent inhibition with lower blocking efficiency when compared to DCPIB at normal resting membrane potentials. Under isotonic conditions and pH 4.5, the cells showed a more inconsistent behavior. Although, on average, the cell volume was relatively constant, we observed ~5% cell swelling in two out of three experiments. We assume that this is the manifestation of impaired cell volume regulation and acidotoxic/necrotic volume increase, similar as that previously described in microglial cells [51], neurons [10], and HeLa cells [5]. The apparently unchanged volume could be the result of an overlapping of cell swelling under acidic conditions and the gradual shrinkage that was observed under normal pH. Cell swelling by acidification might be caused by increased NaCl uptake due to parallel ASOR Cl^−^ current activation and the activation of acid-sensitive cation channels, such as ASICs or TRPM7 channels. Cell swelling under acidic conditions was enhanced by DIDS, which could be due to the inhibition of the residual volume-sensitive Cl^−^ current or other DIDS-sensitive transport mechanisms. This contrasts findings in HeLa cells and cortical neurons, where acidosis-induced swelling was prevented by DIDS and phloretin [5,10]. DCPIB promoted acidotoxic cell swelling during 20–30 min. Thereafter, however, DCPIB caused progressive cell shrinkage. We have no clear interpretation of this phenomenon. We suppose that DCPIB has a cytotoxic, pro-apoptotic effect at pH 4.5 associated with an apoptotic volume decrease (AVD), as we did not see such an effect at normal pH. Under hypotonic conditions and pH 7.4, BV-2 cells accomplished a complete RVD within 10 min., which was equally inhibited by both DCPIB at pH 7.4 and extracellular acidification to pH 4.5. Impaired RVD under acidic conditions might, at least in part, be the consequence of VSOR current inhibition and ASOR current activation. When the cell membrane potential is depolarized close to the reversal potential of Cl^−^ due to Cl^−^ current activation, the relative contribution of Cl^−^ ions to the osmotic driving force for water efflux would be reduced and the efflux of organic osmolytes would remain as the main driving force for the RVD. However, if ASOR channels, in contrast to VSOR channels, were impermeable for organic osmolytes, ASOR current activation would have the same effect as pharmacological inhibition of the VSOR current. In any case, our data imply that cell volume homeostasis in microglial cells is massively impaired under acidic conditions, and that ASOR channels cannot substitute for VSOR channel function in cell volume regulation.

## 4. Materials and Methods

### 4.1. Salts, Chemicals, Drugs

All the salts and chemicals were *p.a.* grade. DIDS, nigericin, and tamoxifen were purchased from Sigma-Aldrich-Merck (Darmstadt, Germany), DCPIB from Tocris-biotechne (Abingdon, UK), and BCECF-AM from Merck-Calbiochem (Darmstadt, Germany). Stock solutions of nigericin (5 mg/mL), tamoxifen (40 mM), and DCPIB (100 mM) were prepared in ethanol. DIDS and BCECF/AM were dissolved in dimethyl sulfoxide (DMSO) to give stock solutions of 100 and 10 mM, respectively. The stocks were stored in aliquots at 20 °C until use.

### 4.2. Cell Culture

The immortal cell line BV-2 (Interlab Cell Line Collection Genova, Genoa, Italy) was grown in RPMI 1640 Medium with stable glutamine and 2.0 g/L NaHCO_3_ (Merck-Biochrom, Darmstadt, Germany; Cat. No. FG 1215; approximate osmolality 290 mOsm/kg) containing 10% fetal bovine serum (FBS, Merck-Biochrom) and 1% antibiotic-antimycotic solution (ABAM, Sigma Aldrich) at 37 °C, 5% CO_2_, and 95% air (standard culture conditions). The subcultures were established once a week by trypsin/EDTA (0.25%; Sigma-Aldrich) treatment.

### 4.3. Patch Clamp

The BV-2 cells were seeded on 0.01% poly-d-lysine (PDL)-coated coverslips (12 mm diameter) and cultured for at least 24 h under standard conditions. The coverslips were transferred to an RC-25 recording chamber (Warner Instruments, Hamden, CT, USA) and then mounted on a Nikon Eclipse TE2000-U inverted microscope. Unless otherwise stated, the experiments were performed at room temperature in the whole-cell perforated patch clamp configuration while using amphotericin B. The recordings were started when the serial resistance was <30 MΩ for the perforated configuration and <10 MΩ if the ruptured configuration was applied. Patch electrode resistances were 4–9 MΩ. Data were recorded while using an EPC-10 amplifier controlled by PatchMaster software (HEKA, Lambrecht/Pfalz, Germany). Cell membrane potential (V_mem_) recordings were performed in the zero-current clamp mode. The intracellular (pipette) solution contained (in mM): 70 K_2_SO_4_, 10 NaCl, 10 KCl, 4 MgCl_2_, 2 CaCl_2_, 5 HEPES free acid, 10 EGTA (249 mOsm/kg, pH 7.2 adjusted with KOH), and 130 µM amphotericin B. The isotonic extracellular control solution contained (in mM): 140 NaCl, 5.6 KCl, 2.5 CaCl_2_, 1.5 MgCl_2_, 10 HEPES free acid, 4.5 glucose, and 5 mannitol (300 mOsm/kg, pH 7.4 adjusted with NaOH). The voltage clamp recordings of ASOR and VSOR currents were performed under symmetrical intra- and extracellular Cl^−^ conditions. The isotonic extracellular control solution consisted of (in mM): 100 NaCl, 2.5 CaCl_2_, 2.5 MgCl_2_, 10 HEPES free acid, and 80 mannitol (300 mOsm/kg, pH 7.2 adjusted with NaOH). Mannitol was omitted to obtain a hypotonic (220 mOsm/kg) solution for VSOR activation. To assess pH dependencies, the extracellular solution was titrated to pH values ranging from 3.0 to 5.0. The pH was adjusted with HCl into a range of ± 0.05 from the intended values immediately prior to use. The pipette solution contained (in mM): 100 CsCl, 5 MgCl_2_, 10 HEPES free acid, 11 EGTA, 60 raffinose, 2 Mg-ATP (303 mmol/kg, pH 7.2 adjusted with CsOH), and 130 µM amphotericin B. The pipette solution was titrated to pH 4.5 (HCl) for recoding the effect of an intracellular acidification. The currents were monitored in response to voltage ramps (500 ms duration, 10-s interval) and voltage steps (500 ms duration, increments of 20 mV) from −100 to +100 mV. The holding potential between the ramps/steps was 0 mV to desensitize the voltage-activated currents. Bath solution exchange was performed with a valve-controlled gravity-driven perfusion system (ALA Scientific Instruments, Farmingdale, NY, USA) at a flow rate of 3–5 mL/min. Osmolalities of intra- and extracellular solutions were measured while using a Wescor^®^ vapor pressure osmometer.

### 4.4. Cell Volume Measurements

The BV-2 cells were harvested by Trypsin/EDTA after growing for 4–5 days under standard conditions. The cell suspension was split into aliquots to measure the effect of extracellular pH. Aliquots were centrifuged for 5 min. at 200× *g* and the supernatants were discarded. The cell pellet was re-suspended in 20 ml of an extracellular solution containing (in mM) 140 NaCl, 5.6 KCl, 2.5 CaCl_2_, 1.5 MgCl_2_, 10 HEPES free acid (FA), 4.5 glucose, and 5 mannitol immediately before the first measurement (time point 0) (300 mOsm/kg), in which the pH was adjusted to pH 7.4, 6.0 or 4.5 with NaOH or HCl. For the pH 4.5 solutions, MES hydrate was used instead of HEPES free acid. The pH of the solutions was titrated into a range of ± 0.05 from the intended value, immediately before use. The hypotonic extracellular solution (220 mOsm/kg) was obtained by a reduction of NaCl to 100 mM. Blockers (DIDS or DCPIB) were added to the samples, as indicated. The mean cell volumes (MCV in fl) in the different samples were alternately measured every 5 min. over 60 min. on a Beckman Coulter Z2 particle counter (Beckman Coulter, Krefeld, Germany). Between two measurements, the aperture tube was rinsed with a solution of the same pH as in the following sample to minimize the pH alterations by liquid carryover. The Coulter method is based on measuring changes in electrical resistance produced by nonconductive particles that were suspended in an electrolyte solution (Coulter method). Calibration for particle size was done while using 10-µm Flow-Check fluorospheres (Beckman Coulter). The data were analyzed with the Multisizer Software (Beckman Coulter) while using a 600-fl cutoff to exclude cell debris.

### 4.5. Intracellular pH (pH_i_) Measurements

Cells were seeded in 96-well black, clear bottom microplates at a density of 1.5 × 10^5^ cells/mL and kept under standard conditions for 24–48 h. The cells were loaded with 5 µM of the membrane permeable pH-sensitive dye BCECF-AM for 30 min. at 37 °C in serum-free medium. After the removal of the loading medium 100 µL of extracellular solution containing (in mM) 140 NaCl, 5.6 KCl, 2.5 CaCl_2_, 1.5 MgCl_2_, 10 HEPES free acid, 4.5 glucose, and 5 mannitol (300 mOsm/kg) with pH being adjusted to 7.4, 6.0, 5.0, or 4.5 were added into the wells in quadruplicates. The pH of the solutions was titrated into a range of ± 0.05 from the intended value immediately before use. The fluorescence measurements (bottom readings) were performed at 37 °C in a humidity cassette on a Spark 20 M multimode plate reader (Tecan, Grödig, Austria). BCECF was alternately excited at 440 and 490 nm while using the built-in monochromator and emission was measured at 535 nm (20-nm bandwidth, 40 µs integration time) every 5 min. over 75 min. Readings from wells containing non-BCECF-loaded cells were used for background subtraction. 490/440 nm ratios calculated from the corrected fluorescence intensity values were converted to absolute pH while using the high K^+^/nigericin calibration method (cells on the same microplate were exposed to solutions titrated to pH 8.0, 7.0, 6.0, or 5.0 in the presence of 140 mM KCl and 10 µg/mL nigericin, for pH interpolation, the 490/440 nm fluorescence ratios that were obtained from these samples were fitted with a second-order polynomial function).

### 4.6. Statistics

Data are expressed as means ± standard error of the means (SEM) of at least three independent biological replicates (*n* ≥ 3). The solvent control samples were included in all experimental series. Statistical analysis was carried out using Student’s *t*-test, Wilcoxon test, or one or two-way ANOVA with Dunnett’s, Tukey’s, or Bonferroni’s tests for multiple comparisons, as applicable. Means were considered to be significantly different at *p*-values < 0.05 (indicated as *, or #). Data were analyzed and plotted while using GraphPad Prism 7 (GraphPad Software, La Jolla, CA, USA) or Igor Pro 6 (WaveMetrics, Portland, OR, USA). 

## 5. Conclusions

We describe an endogenous acid-sensitive outwardly rectifying (ASOR) anion (Cl^−^) current in BV-2 microglial cells, which shares characteristics with ASOR currents in other cell types, including its sensitivity to extracellular but not intracellular acidification, pronounced outward rectification, anion permeability sequence, and pharmacological profile. Our data add to a better understanding of the electrophysiological properties of microglial cells in general and the activation behavior of ASOR channels. We compared the properties of the acid-sensitive current with those of the volume-sensitive outwardly rectifying (VSOR) Cl^−^ current and then worked out an interrelation between these two conductances and possible functional consequences of this interrelation for cell volume homeostasis. We find a short-term coexistence and superimposition of ASOR and VSOR currents under simultaneous acidic and hypotonic conditions, but the ASOR current dominates in the longer term, while the VSOR current gets inactivated. Importantly, we show that the cell volume regulatory ability of BV-2 cells is severely impeded under acidic conditions and conclude that ASOR channels cannot fulfill a function in cell volume regulation. Acidosis is a hallmark of pathophysiological events, like inflammation, stroke, or ischemia. Migration and phagocytosis in the microglial cells closely related to cell volume regulatory processes. Under acidotoxic conditions in the brain, ASOR current activation could therefore affect microglial function.

## Abbreviations

ASOR	Acid-sensitive outwardly rectifying
AVD	Apoptotic volume decrease
BAPTA-AM	1,2-bis(2-aminophenoxy)ethane-N,N,N′,N′-tetraacetic acid tetrakis, acetoxymethyl ester
BCECF-AM	(2’,7’-bis-(2-carboxyethyl)-5-(and-6)-carboxyfluorescein, acetoxymethyl ester)
DCPIB	4-[(2-butyl-6,7-dichloro-2-cyclopentyl-2,3-dihydro-1-oxo-1H-inden-5-yl)oxy]butanoic acid
DIDS	4,4′-diisothiocyanatostilbene-2,2′-disulfonic acid
MCV	Mean cell volume
NPPB	5-nitro-2-(3-phenylpropylamino)-benzoic acid
pH_i_	Intracellular pH
RVD	Regulatory volume decrease
V_mem_	Cell membrane potential
VSOR	Volume-sensitive outwardly rectifying
